# Editorial: Neuronal Development and Degeneration

**DOI:** 10.3389/fgene.2019.01213

**Published:** 2019-11-22

**Authors:** Patrice E. Fort, Nan-Jie Xu, Wenbo Zhang

**Affiliations:** ^1^Department of Ophathalmology and Visual Sciences, University of Michigan, Ann Arbor, MI, United States; ^2^Department of Anatomy and Physiology, Shanghai Jiao Tong University School of Medicine, Shanghai, China; ^3^Department of Ophthalmology & Visual Sciences, University of Texas Medical Branch, Galveston, TX, United States

**Keywords:** neuronal development, degeneration, retina, brain, neurodegenerative disease

This research topic combined exciting new findings in original research articles with well-rounded in-depth reviews of some of the most important mechanisms and pathways involved in two major aspects controlling the central nervous system (CNS)’s function: neurodevelopment and at the other end, neurodegeneration.

Among some of the most burning questions in the field of CNS research are the role of the immune response and the resident immune cells in the development of the central nervous system on one hand, and that of neuroinflammation and its regulation in the context of neurodegenerative conditions on the other. In their original study, Duncan et al. showed a new central role of the chemokine Ccl5 as a mediator of inner retinal circuitry during development using functional, morphometric and immunohistochemical analysis in transgenic mice deficient for this protein. In doing so, the authors nicely showed how lack of this protein led to significant perturbations of the intra-retinal wiring of retinal bipolar and ganglion cells of the retina. While the underlying mechanism and pathways involved remain to be identified, in this special topic, a study by Pozner et al. used inducible pluripotent stem cells (iPSCs)-derived cortical neurons to study the role of GSK3β/βCateninsignaling in the process of neurite growth, especially so in the context of hereditary spastic paraplegias. Using a GSK3β inhibitor and patient derived iPSCs, they showed that mutations in SPG11, the most common genetic cause of this disease, they could restore neurite growth, but also improve overall neuronal health and survival.

In addition to providing interesting tools to study mechanistic aspects of normal and diseased development, studies are being conducted to harness the potential of pluripotent stem cells as a therapeutic option. Rabesandratana et al. put together an exciting and in depth review of the current state of the field of pluripotent stem cells as a mechanistic tool but also as an avenue for the treatment of optic neuropathies. The authors are nicely summarizing the recent advancement of this field relative to the production, characterization and delivery of iPSCs-derived retinal ganglion cells, highlighting the potential of the field but also its remaining challenges.

Another group of publications in this research topic focused on different neuroprotective pathways and their role in normal neurodevelopment as well as in neurodegenerative conditions. An in depth review by Miller and Fort nicely summarizes the current knowledge relative to the role and function of heat shock proteins in neurodevelopment, to put in prospective with their better known role in neuroprotection. In this review, the authors highlight the critical roles that these chaperone proteins play in the regulation of neuronal and glial maturation by way of regulation of several developmental pathways. This research topic also includes 3 original research articles pertaining to the topic of neuroprotective molecular mechanisms. One of them is exploring more in depth the mechanisms of action of the well-recognized antioxidant and pro-survival transcription factor NRF2, and reports the discovery of its regulation by the ER stress related factor XBP-1. The authors of this manuscript nicely demonstrate this regulation using loss and gain of function approaches in primary retinal pigment epithelial (RPE) cells (Chen et al.). Focusing on the excitotoxicity-induced neurodegenerative model, another group reported the potential of polyamine oxidase as a therapeutic target. This study reports that a systemically administered polyamine oxidase inhibitor is associated with a significant improvement of ganglion cell survival, suggesting a role for this enzyme in the regulation of pro-survival signaling pathways (Pichavaram et al.). In a separate manuscript, the same group used a loss of function approach to show an important role of another enzyme, Arginase 2, in the regulation of axonal injury. In this work, the authors have gathered data suggesting that this effect is due to its role in regulating the potent growth factor BDNF concomitantly with a reduction of the injury-associated inflammation/glial activation (Xu et al.).

This paper by Xu et al. interestingly substantiate the review by Ngwenya and Danzer, which focused on the consequences of traumatic brain injury (TBI) on hippocampal change, and the relationship to adult neurogenesis. The authors further discuss how current treatments for TBI can also alter adult neurogenesis, and the dire need for less neurogenesis destabilizing new treatments for TBI. Another study looked at the impact of diffuse axonal injury in corpus callosum and brain stem, once again emphasizing the role of inflammation and glial dysfunction/activation in the progressive degeneration. The main finding of this study was the difference in pathophysiology between those brain regions, and the distinct processes of myelin disruption and axonal degeneration (Mu et al.).

When neurodegeneration could not be prevented, regeneration is the remaining option. In their review, Zhang et al. report on the complexity of this approach and the recent realization of the need for a coordination of multiple inhibitory and permissive signals involving the central phosphatase PTEN.

Finally, this research topic includes manuscript relative to developmental and aging brain disorders, including Autism Spectrum Disorder (ASD) and Alzheimer’s disease (AD), and some of the new findings obtained by multiple approaches focusing on human tissue analysis: genetic, histopathologic, and primary cell culture and transcriptomic. Du et al. demonstrated that whole exome sequencing could be an effective method for early diagnose of ASD, especially those with negative findings of copy number variants. A separate manuscript of this research topic reports the results of a novel study of the relationship between brain region volume and polygenic risk factor in the brain of patients with AD, which identified a specific region of the brain to be associated with polygenic risk factor (Wang et al.). In a separate perspective article, Zhao et al. focused on the role of miRNA in the pathogenic mechanisms of AD, and how these miRNA have been studied in primary neuroglial cells isolated from AD and normal donors to assess their role in regulation of the neuroglial transcriptome and more specifically the control of synaptogenesis. These miRNA have received significantly more attention since the discovery of their increased abundance in the neocortex of patients with sporadic AD associated with the down-regulation of critical brain-specific genes.

Altogether, the papers published in this special research topic of Frontiers in Neuroscience clearly support the emerging paradigm of interconnection between neurodegeneration and neuroinflammation on one side and normal neurodevelopment and disease mechanisms on the other. It also emphasize the need for a general understanding of normal physiological mechanisms in order to define pathophysiological ones and develop the knowledge necessary for the identification and characterization of new therapies for neurodegenerative conditions.

## Author Contributions

PF wrote the manuscript, and PF, N-JX and WZ edited the manuscript.

## Funding

Supported by National Institutes of Health grants EY020895 (PF), EY022694, EY026629 and Retina Research Foundation (to WZ), and National Natural and Science Foundation of China 31671062 and 81870820 (to N-JX).

## Conflict of Interest

The authors declare that the research was conducted in the absence of any commercial or financial relationships that could be construed as a potential conflict of interest.

